# Malignant transformation of a gastric hyperplastic polyp in a context of *Helicobacter pylori*-negative autoimmune gastritis: a case report

**DOI:** 10.1186/s12876-016-0537-x

**Published:** 2016-10-12

**Authors:** Kenichi Yamanaka, Hiroyuki Miyatani, Yukio Yoshida, Takehiro Ishii, Shinichi Asabe, Osamu Takada, Mitsuhiro Nokubi, Hirosato Mashima

**Affiliations:** 1 Department of Gastroenterology, Jichi Medical University, Saitama Medical Center, Saitama, 330-8503 Japan; 2 Department of Surgery, Jichi Medical University, Saitama Medical Center, Saitama, 330-8503 Japan; 3 Department of Pathology, Jichi Medical University, Saitama Medical Center, Saitama, 330-8503 Japan

**Keywords:** Gastric hyperplastic foveolar polyp, Autoimmune gastritis, *Helicobacter pylori*, Gastric carcinoma, Case report

## Abstract

**Background:**

Gastric foveolar hyperplastic polyps (GFHPs) are common findings in clinical practice. GFHPs commonly arise in a background of chronic atrophic gastritis, including autoimmune gastritis (type A gastritis), and have a potential risk of malignant transformation.

**Case presentation:**

In 2005, a 55-year-old Japanese woman underwent upper endoscopy at another hospital and was found to have a pedunculated polyp (10 mm in diameter) on the greater curvature of the lower gastric body. On biopsy, the polyp was diagnosed as a GFHP. Nine years later, the polyp had grown to 20 mm in diameter, and the biopsy specimen taken at this time showed tubular adenocarcinoma. On admission to our hospital, the serum *Helicobacter Pylori* (*H. pylori)* immunoglobulin G antibody and stool *H. pylori* antigen were both negative. Anti-gastric parietal cell antibody was positive, as was the anti-intrinsic factor antibody, and the fasting serum gastrin level was markedly increased. In 2014, en bloc resection of the pedunculated polyp was performed by endoscopic submucosal dissection. The final histological diagnosis was adenocarcinoma of the stomach with submucosal and lymphatic invasion. Subsequently, additional radical distal gastrectomy was performed. At the latest follow-up (12 months postoperatively), no recurrence was noted.

**Conclusions:**

We here reported a rare case of malignant transformation of GFHP arising in a context of type A gastritis. To our knowledge, there are no previous reports on malignant transformation of GFHP with submucosal and lymphatic invasion arising in a background of type A gastritis in the English literature. Further, there is currently no effective treatment other than endoscopic or surgical treatment for such cases. Given the potential risk of malignant transformation due to hypergastrinemia, we consider that endoscopic treatment should be considered as a first-line therapy when a malignant growth is suspected.

## Background

Gastric foveolar hyperplastic polyps (GFHPs) are common findings in clinical practice. They do not regress or disappear spontaneously and are associated with a risk of malignant transformation in 0.6–4.5 % of cases [1–5]. GFHPs commonly arise in a background of chronic atrophic gastritis, including both type B gastritis, associated with *Helicobacter Pylori* (*H. pylori*) infection, as well as type A gastritis (autoimmune gastritis) [6]. In Japan, type B gastritis accounts for > 76 % of all gastritis cases [7, 8]. A previous study reported that eradication of *H. pylori* led to regression and disappearance of gastric hyperplastic polyps in approximately 70 % of patients [9]. In this context, *H. pylori* eradication therapy is recommended in Japan. However, *H. pylori* eradication therapy is not effective for all cases, particular for GFHPs arising in a background of autoimmune gastritis (type A gastritis). With the increased prevalence and effectiveness of *H. pylori* eradication therapy, the clinical impact of autoimmune gastritis, and thereby also of GFHPs, is rising. There is currently no effective treatment other than endoscopic or surgical treatment for GFHPs arising in a background of *H. pylori*-negative type A gastritis. Therefore, endoscopic treatment is recommended as the first-line therapy when a malignant growth is suspected.

## Case presentation

**Fig. 1:**
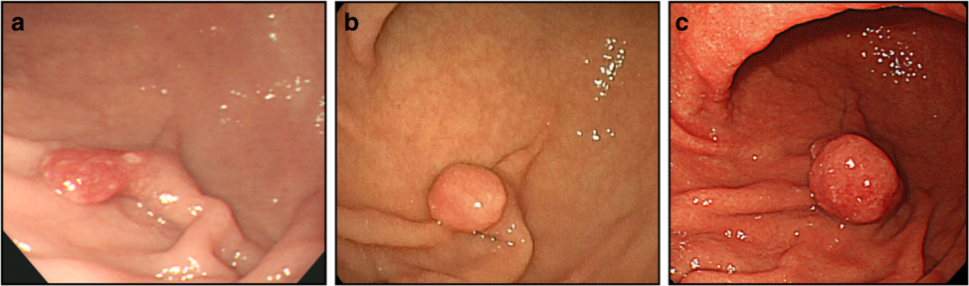
Endoscopy findings. **a**. In 2005, a pedunculated polyp measuring approximately 10 mm in diameter was observed on the greater curvature of the lower gastric body. The surface of the polyp was slightly reddish. On biopsy, the lesion was diagnosed as a hyperplastic foveolar polyp. **b**. In 2013, the pedunculated polyp on the greater curvature of the lower gastric body had grown to 12 mm in diameter. The head of the polyp was more rounded compared to the image taken in 2005. **c**. In 2014, the pedunculated polyp on the greater curvature of the lower gastric body had grown to 20 mm in diameter. The head of the polyp was slightly reddish, more rounded, and tense

In November 2005, a 55-year-old Japanese woman underwent upper endoscopy at another hospital and was found to have a pedunculated polyp, measuring 10 mm in diameter, on the greater curvature of the lower gastric body (Fig. 1a). On biopsy, it was diagnosed as a GFHP. At the moment of the first diagnosis, there was only an endoscopic report and the endoscopic appearance could be compatible with an atrophic mucosa. In January 2013, upper endoscopy was repeated, which revealed that the polyp had grown to 12 mm in diameter with a slightly rounded head (Fig. 1b). Biopsy was not performed at the time.

**Fig. 2:**
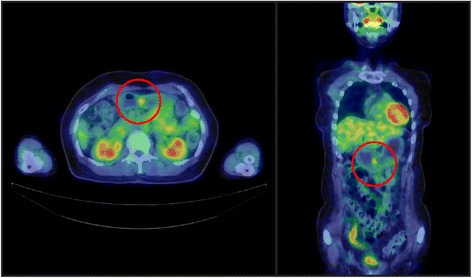
Positron emission tomography-computed tomography findings. A well-circumscribed mass was observed in the lower gastric body, with a maximum standardized uptake value of 2.7

In June 2014, the patient underwent positron emission tomography-computed tomography to evaluate the treatment effect on her right breast cancer, for which she had undergone surgery and received chemotherapy and radiation therapy at the age of 53 years. There was an abnormal accumulation in the lower gastric body (Fig. 2). Subsequent upper endoscopy showed that the polyp had grown to 20 mm in diameter, and the surface of the head was slightly reddish and tense (Fig. 1c). The biopsy specimen obtained from the head of the polyp was histologically diagnosed as tubular adenocarcinoma and was determined to be unrelated to the breast cancer. It was suggested that the polyp became malignant during the nine years of follow-up, and the patient was referred to our institution for further evaluation and treatment.

### Physical examination

On physical examination, scars from the right breast mastectomy with bilateral axillary lymph node dissection were noted. No other remarkable findings were observed.

### Laboratory data (Table 1)

**Table 1:** Laboratory data

Hematological analysis	Value	Unit	Blood chemistry	Value	Unit
White blood cell count	4800	/μl	TP	7.3	g/dl
Hemoglobin	11.9	g/dl	Alb	4.4	g/dl
Hematocrit	37.8	%	T-bil	0.81	mg/dl
Platelets	19.5 × 10^4^	/μl	D-bil	0.24	mg/dl
			AST	34	U/L
Tumor markers			ALT	25	U/L
Carcinoembryonic antigen	1.3	ng/ml	LDH	197	U/L
Cancer antigen-15-3	17.1	U/ml	γ-GTP	16	IU/l
			BUN	11	mg/dl
Anti‐parietal cell antibody	80	fold	Cre	0.55	mg/dl
Anti-intrinsic factor antibody	+		Gastrin	>3000	pg/dl
Pepsinogen I	7.6	ng/ml	Vit B12	208	pg/ml
Pepsinogen II	16.3	ng/ml			
Pepsinogen I/II/ ratio	<0.5	ng/ml			

*Abbreviations*: *TP* total protein, *Alb* albumin, *T-bil* total bilirubin, *d-bil* direct bilirubin, *AST* aspartate aminotransferase, *ALT* alanine aminotransferase, *LDH* lactate dehydrogenase, *γ-GTP* gamma-glutamyl transpeptidase, *BUN* blood urea nitrogen, *Cre* creatinine, *Vit* vitamin

No abnormalities were found in the blood counts or liver and renal function tests. The serum *H. pylori* immunoglobulin G antibody and stool *H. pylori* antigen were both negative. The anti-gastric parietal cell antibody was positive (80-fold increase), as was the anti-intrinsic factor antibody. The fasting serum gastrin level was markedly increased at > 3000 pg/ml (normal range, < 200 pg/ml). The serum pepsinogenI level and pepsinogen I/II ratio were both low (Table 1).

### Medication

The patient was not taking any medication at the time.

### Positron emission tomography-computed tomography (July 2014) (Fig. 2)

A well-circumscribed mass with a maximum standardized uptake value of 2.7 was observed in the lower gastric body. There were no findings suggesting the presence of gastrinoma.

### Clinical course

**Fig. 3:**
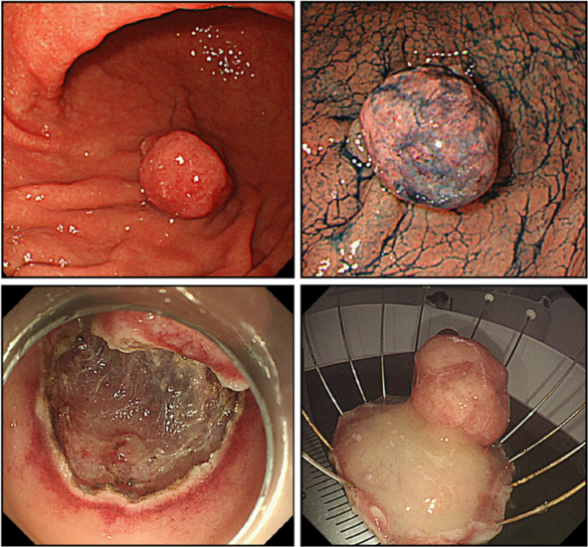
Endoscopic submucosal dissection. En bloc endoscopic submucosal dissection was performed for the pedunculated polyp, measuring 20 mm in diameter, on the greater curvature of the lower gastric body

In August 2014, en bloc resection of the pedunculated polyp, measuring 20 mm in diameter, on the greater curvature of the lower gastric body was performed by endoscopic submucosal dissection (Fig. 3).

**Fig. 4:**
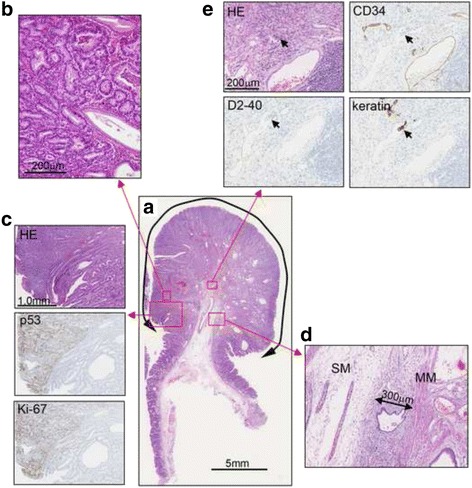
Histological findings. **a**. Hematoxylin and eosin (HE) staining. **b**. High magnification image of the boxed area. **c**. HE, p53, and Ki-67 stainings of the border between the hyperplastic glands and the tumor glands. The tumor lesions were strongly positive for p53 and Ki-67. **d**. Submucosal invasion in the stalk, with an invasion depth of 300 μm (MM, muscularis mucosae; SM, submucosal layer). **e**. HE, cluster of differentiation 34 (CD34), D2-40, and keratin staining. In CD34-negative and D2-40-positive lymph ducts, keratin-positive cells with acidophilic cytoplasm and large nuclei were observed, indicating lymphatic invasion (*arrows*)

Histopathological examination showed tumor lesions, which had well-defined borders with hyperplastic glands (Fig. 4a, b). The tumor lesions were strongly positive for p53 and Ki-67, while the hyperplastic lesions were not (Fig. 4c). Submucosal invasion was observed in the stalk, with an invasion depth of 300 μm (Fig. 4d). In D2-40-positive and cluster of differentiation 34-negative lymph ducts, cells with acidophilic cytoplasm and large nuclei were observed. The cells were positive for keratin, indicating lymphovascular invasion (Fig. 4e). As a result the final histological diagnosis was adenocarcinoma of the stomach, tubular adenocarcinoma > papillary adenocarcinoma, size of resected lesion 20 mm in size, submucosal invasion of 0.3 mm, venous invasion (-), lymphatic invasion (+).

**Fig. 5:**
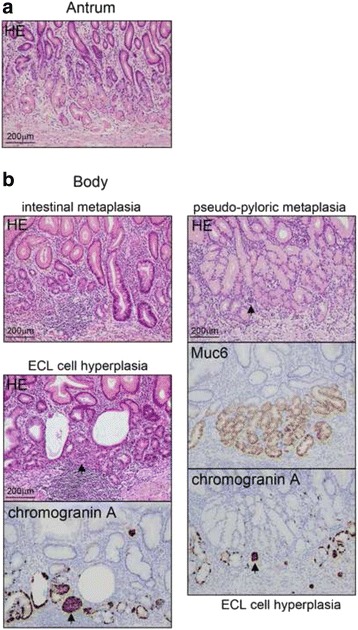
Examination of the background mucosa. **a**. The proper gastric glands in the pyloric region were preserved. There was no atrophic change. **b**. The proper gastric glands in the gastric body. The mucosa was atrophic and showed intestinal metaplasia and pseudo-pyloric metaplasia. Muc6 is a gastric pyloric gland- type secretory mucin. Enterochromaffin-like cell hyperplasia (linear hyperplasia and micro-nodular hyperplasia (*arrows*)) was revealed by chromogranin A staining

Consequently, additional radical distal gastrectomy was performed. Examination of the surgical specimens showed a scar resulting from the endoscopic submucosal dissection on the anterior wall of the gastric body. There were no residual tumor cells or lymph node metastasis. Examination of the background mucosa showed that the proper gastric glands in the pyloric region were preserved, while those in the mucosa of the gastric body were atrophic and showed intestinal metaplasia and pseudo-pyloric metaplasia (Fig. 5). In the OLGA staging system, the corpus and the antrum were scored as 2 and 0, respectively, indicating stageII gastritis at the time of surgery [10, 11]. Chromogranin A staining revealed enterochromaffin-like cell hyperplasia (linear hyperplasia and micronodular hyperplasia) (Fig. 5b, arrows indicate micronodular hyperplasia). *H. pylori* infection was negative (Fig. 5).

## Conclusions

The classification of chronic gastritis into types A and B was proposed by Strickland et al. [12]. However, currently, there are no established diagnostic criteria for the diagnosis of type A gastritis. Traditionally, type A gastritis has been diagnosed based on the anti-gastric parietal and anti-intrinsic factor antibody findings, presence of corpus-predominant atrophic gastritis, and serum gastrin levels, with Ban et al. stating that asymptomatic type A gastritis could be diagnosed by anti-gastric parietal antibody [13]. The prevalence of autoimmune gastritis is unclear [13, 14]. In the early stage, the diagnosis of type A gastritis is difficult even with a biopsy, since the symptoms of anemia due to malabsorption of vitamin B12 and iron usually develop at a later stage. In the present case, we considered that the patient was not infected with *H. pylori*, because her serum *H. pylori* immunoglobulin G antibody, stool *H. pylori* antigen, and histological examination were all negative. On the other hand, the patient was positive for anti-gastric parietal cell and anti-intrinsic factor antibodies, and had a markedly elevated level of serum gastrin and reduced pepsinogenI level and pepsinogenI/II ratio. Moreover, the patient had corpus-predominant atrophic gastritis, OLGA stageII, and enterochromaffin-like cell hyperplasia. Taken together, these findings led to the diagnosis of type A chronic atrophic gastritis. Although we did not have the sufficient histological data from the first endoscopy performed in 2005, mild corpus atrophy was detected at that time. Rugge et al. reported that in 116 patients with autoimmune gastritis, 87 cases (75.0 %) remained and 25 cases (21.6 %) progressed to a higher OLGA gastritis stage during a mean follow-up period of 54 months (range, 24–108 months) [11]. Considering the gastritis stage in 2014, the present case can thus be speculated to have been at stageI orII in 2005. Type A gastritis is characterized by the presence of hypergastrinemia associated with reduced gastric acid secretion. Gastrin is a peptide hormone synthesized and released mainly from G cells in the gastric antrum, which plays a role in the regulation of gastric acid secretion and gastrointestinal motility [15]. Furthermore, it is known to exert trophic actions on digestive cells [16, 17]. In the present patient, it was suggested that the GFHP had grown during the nine-year follow-up period, owing to her hypergastrinemia; her serum gastrin level was > 3000 pg/ml before undergoing distal gastrectomy, but decreased to 74 pg/ml 48 h after the surgery.

The process of cancer development is considered to be a multistage process (hyperplasia ➔ dysplasia ➔ carcinoma); that is, as the polyp grows and becomes more dysplastic, the risk of cancer increases [2, 5, 18, 19]. The most important risk factors of malignant transformation in chronic atrophic gastritis include age over 50 years, severe atrophy, and the presence of intestinal metaplasia extension [20]. The present patient fit the first and third risk factors. Rugge et al. reported that the risk of cancer was restricted to case of high-risk gastritis stage (OLGA stages III-IV) and that it was associated mainly with concomitant *H. pylori* infection [11]. However, malignant transformation in type A gastritis associated with hypergastrinemia might be induced at an earlier gastritis stage. Several genetic alterations have been revealed in hyperplastic polyps, including abnormal expression of tumor suppressor p53, K-ras mutations, and microsatellite instability [18, 21]. Shibahara et al. conducted a study of gastric hyperplastic polyps and reported that increased expression of p53 was observed in dysplastic and carcinoma cells but not in the normal or hyperplastic cells, and that the expression of Ki67 was higher in the carcinoma and dysplastic cells than in the hyperplastic cells. They suggested that examinations of p53 and Ki67 were useful markers of malignant transformation of gastric hyperplastic polyps [22]. Yao et al. and Zea-Iriarte et al. also suggested that p53 plays an important role in the malignant transformation of gastric hyperplastic polyps [19, 23]. In the present case, the expression of p53 was strongly positive in the carcinoma cells and Ki67 was positive in approximately 50 % of the carcinoma cells, while these stainings were much decreased in the non-transformed cells (Fig. 4b).

The reported incidence of malignant transformation of GFHP ranges from 0.6 to 4.5 % [1–5], and Fujino et al. reported that the mean diameter of GFHPs with malignant transformation was as large as 23.4 ± 10.2 mm [24]. Regarding the histological type, most previously reported cases were differentiated adenocarcinomas, while a few were poorly-differentiated adenocarcinomas or signet ring cell carcinomas [25–28]. The GFHP in the present patient was 20 mm in diameter and histologically diagnosed as differentiated adenocarcinoma (Fig. 4).

The diagnostic criteria of malignant GFHP proposed by Nakamura et al. in 1985 are as follows [29]: 1) coexistence of benign and malignant parts in the same polyp; 2) existence of sufficient evidence that the benign area has previously been a benign polyp; and 3) existence of sufficient cellular and structural atypia in the malignant area to be diagnosed as cancer. The patient in the present study met these criteria and was therefore diagnosed with malignant transformation of GFHP over nine years. GFHPs with carcinoma have been reported to have the following macroscopic characteristics: pedunculated form, larger size (>20 mm), reddish color, irregular or nodular surface, white-coated, and bleeding tendency [2, 23, 25, 30, 31]. Our patient displayed four of these macroscopic features, including pedunculated form, a diameter > 20 mm, growth tendency, and a white-coated lesion.

It has been reported that magnifying endoscopy with narrow band imaging is useful for the diagnosis of cancer [32–34]; however, it is not sufficiently useful for diagnosing malignant transformation of gastric hyperplastic polyps [23]. Ahn et al. reported that GFHPs measuring 10 mm or larger in diameter should be endoscopically resected [35], whereas Tanabe et al. reported the disappearance of a 20 mm hyperplastic polyp with malignant transformation after *H. pylori* eradication therapy [36]. The lesion in the present patient was diagnosed as tubular adenocarcinoma on biopsy. Endoscopic estimation of the invasion depth of the carcinoma was difficult; hence, endoscopic submucosal dissection, a minimally invasive procedure, was performed. The results showed a submucosal invasion depth of 0.3 mm and lymph duct invasion. Subsequently, we performed additional distal gastrectomy and lymph node dissection. There was no distant metastasis, and we considered that complete removal of the tumor was achieved. At the time of this report, the patient has been followed-up for 12 months after surgery with no recurrence.

To our knowledge, there are only a few previous cases, limited to Japan, of malignant GFHP with submucosal invasion requiring surgical treatment [37, 38], and there are no previous reports on malignant transformation of GFHP with submucosal invasion and lymphatic invasion arising in a background of type A gastritis in the English literature. Thus, the present case is considered very rare and to provide valuable information. Importantly, there is currently no effective treatment other than endoscopic or surgical treatment for GFHPs, secondary to type A gastritis. Moreover, as this type of polyp has a potential risk of malignant transformation due to hypergastrinemia, endoscopic or surgical treatment should be considered when malignant transformation is suspected.

## Abbreviation

GFHP: Gastric foveolar hyperplastic polyp
